# Smoking behaviour among children and adolescents in Germany. Results of the cross-sectional KiGGS Wave 2 study and trends

**DOI:** 10.17886/RKI-GBE-2018-025

**Published:** 2018-03-15

**Authors:** Johannes Zeiher, Anne Starker, Benjamin Kuntz

**Affiliations:** Robert Koch Institute, Berlin, Department of Epidemiology and Health Monitoring

**Keywords:** SMOKING, TOBACCO USE, CIGARETTES, HEALTH MONITORING, KIGGS

## Abstract

Smoking behaviour during adolescence is particularly important because the pattern of a person’s tobacco consumption in later life usually is established in this period. According to recent data from KiGGS Wave 2, 7.4% of 11 to 17 year-old girls and 7.0% of boys of the same age smoke at least occasionally. The proportion of children and adolescents who smoke increases with age. Adolescents with high socioeconomic status smoke less frequently than their peers with medium or low socioeconomic status. Since the beginning of the first KiGGS study (2003-2006), the proportion of 11 to 17 year-olds who smoke fell from 21.4% to 12.4% (2009-2012) and has recently dropped to 7.2% (2014-2017). Despite considerable progress, however, there is still potential to improve tobacco prevention policy in Germany for example using taxation and advertising bans.

## Background

Despite a decline in tobacco consumption in almost all industrialised countries, smoking remains the leading cause of premature mortality [1]. About one quarter of adults throughout the world use tobacco, and smoking is also widespread among adolescents [2]. Smoking promotes the development of severe illnesses like cancer, cardiovascular and respiratory diseases [3]. Every year around 7.2 million people die as a result of tobacco smoke – this amounts to approximately 19,600 deaths per day [4]. According to current calculations, there were around 121,000 tobacco-related deaths in Germany in 2013 (13.5% of all deaths in the country) [3].

The population’s tobacco use, therefore, represents a priority from a public health perspective. The smoking behaviour of children and young people is particularly important because smoking uptake usually takes place before the age of 18 [5]. Smokers who start smoking at an early age have an increased risk of developing smoking-related diseases. This is because the organism of adolescents is particularly susceptible to damage by the toxic substances contained in tobacco smoke [5]. Furthermore, people who begin to smoke at an early age also have a lower chance of successfully quitting smoking in later life, this is partly because they are also more likely to become dependent on tobacco [6].

A number of factors influence whether young people actually start smoking. Young people are more likely to smoke if their parents, siblings or peers do so. At the same time, they are more likely to smoke if they are exposed to tobacco advertising, if tobacco use is deemed socially acceptable and when tobacco products are cheap and readily accessible [3].

**KiGGS Wave 2**

Second follow-up to the German Health Interview and Examination Survey for Children and Adolescents

**Data owner:** Robert Koch Institute

**Aim:** Providing reliable information on health status, health-related behaviour, living conditions, protective and risk factors, and health care among children, adolescents and young adults living in Germany, with the possibility of trend and longitudinal analyses

**Study design**: Combined cross-sectional and cohort study

**Cross-sectional study in KiGGS Wave 2**

**Age range:** 0-17 years

**Population:** Children and adolescents with permanent residence in Germany

**Sampling:** Samples from official residency registries - randomly selected children and adolescents from the 167 cities and municipalities covered by the KiGGS baseline study

**Sample size:** 15,023 participants

**KiGGS cohort study in KiGGS Wave 2**

**Age range:** 10-31 years

**Sampling:** Re-invitation of everyone who took part in the KiGGS baseline study and who was willing to participate in a follow-up

**Sample size:** 10,853 participants

**KiGGS survey waves**

► KiGGS baseline study (2003-2006), examination and interview survey
► KiGGS Wave 1 (2009-2012), interview survey
► KiGGS Wave 2 (2014-2017), examination and interview survey

More information is available at www.kiggs-studie.de/english

Over the last 20 years, Germany has stepped up its tobacco prevention policy and implemented various measures aimed at reducing the population’s tobacco consumption, in particular, preventing the uptake of smoking in young people [7]. These measures include the implementation of significant tax increases between 2002 and 2005, non-smoker protection laws at the national and federal-state level and raising the age limit for purchasing and using tobacco products from 16 to 18. Furthermore, they have been accompanied by various setting-based prevention campaigns and programs.

In addition, the Framework Convention on Tobacco Control (FCTC) is an international agreement that was negotiated between numerous countries under the auspices of the World Health Organization and was ratified by Germany in 2003. One of the FCTC’s key aspects is a catalogue of measures that signatory states are required to put in place to reduce the population’s tobacco use [3].

In Germany, these measures are being implemented within the framework of the national health target ‘Reduction of tobacco consumption’; this target was introduced in 2003, evaluated in 2009 and last revised in 2015 [8]. In the context of adolescent smokers, the target focuses on ensuring that teenagers and young adults do not take up smoking and on protecting the population from passive smoking.

In order to be able to review these targets, however, it is essential that reliable, representative data is collected regularly on the smoking behaviour of children and adolescents in Germany. This is done by studies such as the second wave of the German Health Interview and Examination Survey for Children and Adolescents (KiGGS).

## Indicator and methodology

KiGGS forms part of the health monitoring program undertaken at the Robert Koch Institute and includes repeated cross-sectional surveys of children and adolescents aged between 0 and 17 years that are representative of the German population (KiGGS cross-sectional study). After having carried out the baseline study as an interview and examination survey between 2003 and 2006, and KiGGS Wave 1 as an interview-based survey between 2009 and 2012, KiGGS Wave 2 was implemented between 2014 and 2017 as a combined interview and examination survey. A detailed description of the methodology used in KiGGS Wave 2 can be found in New data for action. Data collection for KiGGS Wave 2 has been completed in issue S3/2017 as well as KiGGS Wave 2 cross-sectional study – participant acquisition, response rates and representativeness in issue 1/2018 of the Journal of Health Monitoring [9, 10].

Data on the smoking behaviour of 11 to 17 year-old girls and boys were collected for KiGGS Wave 2 using written questionnaires and information provided by the respondents. The questionnaire included the question, ‘Do you currently smoke?’. The response categories were ‘No’, ‘Daily’, ‘Several times a week,’ ‘Once a week’ and ‘Less [than once a week]’. The KiGGS baseline survey used the same approach to gather data on a participant’s smoking habits [11]. In contrast, KiGGS Wave 1 was conducted by telephone and participants were asked, ‘Have you ever smoked?’ (Answer categories were: ‘Yes’ and ‘No’). If a participant stated that they had smoked at some point in their life, this was followed up by the question ‘How often do you smoke at the moment?’. The answers categories were very similar to those used during the other survey waves ‘Daily’, ’Several times a week’, ‘Once a week’, ‘Less than once a week’ and ‘Not at all’ [12]. In the following, all respondents who stated that they smoke tobacco – including only occasionally – are grouped together as ‘current smokers’. The following also provides details of the prevalence of daily smoking.

The latest KiGGS data is based on information collected from 5,747 adolescents (2,996 girls and 2,751 boys) aged between 11 and 17 years-of-age with valid data on smoking behaviour. The results are presented as prevalences (frequencies) and are stratified by gender, age and socioeconomic status (SES) [13].

The calculations were carried out using a weighting factor that corrected for deviations within the sample from the population structure with regard to age in years, gender, federal state, nationality and the parents´ level of education (Microcensus 2013 [14]). In addition, the calculation of trends over time between the KiGGS waves is based on prevalences that were age-standardised according to the structure of the German population as of 31 December 2015.

This article reports prevalences with 95% confidence intervals (95% CI). A statistically significant difference between groups is assumed to have been demonstrated with p-values of less than 0.05 (once weighting had been applied and the survey design had been taken into account).

## Results and discussion

According to data from KiGGS Wave 2, 7.2% of 11 to 17 year-old children and adolescents in Germany smoke, about half of them do so daily (3.7% of 11 to 17 year-olds). No significant differences were identified according to gender (Table 1). The proportion of children and adolescents who smoke increases steadily with age: whereas less than 1% of girls and boys aged between 11 and 13 smoke at least occasionally, this rate increases among 14 to 17 year-olds to more than 11% (Table 1). The proportion of children and young people who smoke is also related to the socioeconomic status of their family of origin. Both girls and boys from the high status group smoked less frequently than their peers from families with a medium or low socioeconomic status (Table 1).

**Figure 1:**
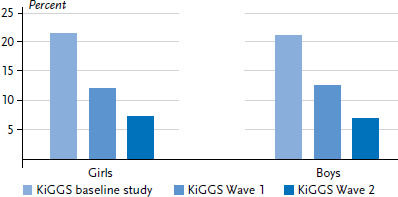
Trends in current smoking among 11 to 17 year-old girls and boys (KiGGS baseline study n=6,729, KiGGS Wave 1 n=4,944, KiGGS Wave 2 n=5,747) Source: KiGGS baseline study (2003-2006), KiGGS Wave 1 (2009-2012), KiGGS Wave 2 (2014-2017)

During the course of the KiGGS study, the proportion of children and adolescents who smoke has fallen sharply. Whereas 21.4% of 11 to 17 year-olds still smoked when the baseline study was conducted (2003-2006), the proportion of smokers had almost halved (to 12.4%) by the time the first follow-up survey was undertaken (2009-2012). Moreover, the proportion of smokers has since fallen to 7.2% (Figure 1). The developments shown by the KiGGS study are in line with findings from other studies in Germany [15]. According to data from the representative surveys conducted by the Federal Centre for Health Education, the proportion of 12 to 17 year-olds who smoke decreased from 22.5% to 7.8% between 2003 and 2015 [16]. In line with the presented results previous KiGGS waves [17] and other studies [18] show that lower rates of smokers are found among children and adolescents with a high socioeconomic status than among their socially disadvantaged peers. Furthermore, clear differences have been found depending on the type of secondary school that young people attend: grammar school students smoke less frequently than pupils from high schools, comprehensives or other secondary school forms [15, 19].

Interpretations of the KiGGS data need to take into account the fact that the study uses information provided by the respondents. Therefore, the results may have been distorted by the provision of socially desirable responses, as respondents may tend to provide what they view as the most socially acceptable answer. This would cause the data to underestimate the actual proportion of smokers [17].

Despite the desirable trend towards fewer adolescents starting to smoke, there is still room for improvement with regard to tobacco prevention policy in Germany. In comparison to other European countries, Germany particularly lags behind in terms of consistently protecting non-smokers from second-hand smoke, as well as in terms of tobacco taxation and the implementation of extensive advertising bans on tobacco products [20].

## Key statements

- The latest data from KiGGS Wave 2 show that 7.2% of adolescents between 11 and 17 smoke and that around half of them do so daily.
- There are no significant differences between girls and boys in terms of smoking behaviour.
- Since the beginning of the KiGGS study (2003-2006), the proportion of 11 to 17 year-olds who smoke has steadily declined from 21.4% to 7.2% (2014-2017).
- Lower smoking rates were found among children and adolescents with high socioeconomic status compared to their peers with low or medium socioeconomic status.

## Figures and Tables

**Table 1:** Prevalence of current and daily smoking according to gender, age and socioeconomic status (n=2,996 girls, n=2,751 boys) Source: KiGGS Wave 2 (2014-2017)

	Current smoking (daily or occasionally)		Daily smoking	
	%	(95% CI)	%	(95% CI)
**Girls (total)**	**7.4**	**(6.2-8.9)**	**3.6**	**(2.8-4.7)**
**Age**				
11-13 Years	0.6	(0.2-1.6)	0.1	(0.0-0.4)
14-17 Years	11.9	(9.9-14.2)	5.9	(4.6-7.6)
**Socioeconomic status**				
Low	9.2	(6.0-13.9)	5.8	(3.5-9.3)
Medium	7.6	(6.2-9.4)	3.4	(2.5-4.7)
High	4.3	(2.6-7.0)	1.5	(0.7-3.0)
**Boys (total)**	**7.0**	**(5.9-8.2)**	**3.9**	**(3.0-5.0)**
**Age**				
11-13 Years	0.9	(0.3-2.8)	0.5	(0.1-3.4)
14-17 Years	11.1	(9.4-13.0)	6.1	(4.7-8.0)
**Socioeconomic status**				
Low	6.7	(4.2-10.4)	2.7	(1.3-5.3)
Medium	8.2	(6.7-10.1)	4.9	(3.6-6.7)
High	3.7	(2.3-5.9)	1.9	(0.9-3.6)
**Total (girls and boys)**	**7.2**	**(6.3-8.2)**	**3.7**	**(3.1-4.5)**

CI=confidence interval
